# Reliability of GENEActiv accelerometers to estimate sleep, physical activity, and sedentary time in children

**DOI:** 10.1186/s12966-021-01143-6

**Published:** 2021-06-06

**Authors:** Devan Antczak, Chris Lonsdale, Borja del Pozo Cruz, Philip Parker, Taren Sanders

**Affiliations:** 1 Institute for Positive Psychology and Education, Australian Catholic University, PO Box 968, Level 10 33 Berry Street, New South Wales 2060 North Sydney, Australia; 2 Institue of Sport Sciences and Clinical Biomechanics, University of Southern Denmark, Odense, Denmark

**Keywords:** Movement Behaviour, Objective Measurement, Youth

## Abstract

**Background:**

Reliable estimates of habitual sleep, physical activity, and sedentary time are essential to investigate the associations between these behaviours and health outcomes. While the number of days needed and hours/day for estimates of physical activity and sedentary time are generally known, the criteria for sleep estimates are more uncertain. The objective of this study was to identify the number of nights needed to obtain reliable estimates of habitual sleep behaviour using the GENEActiv wrist worn accelerometer. The number of days to obtain reliable estimate of physical activity was also examined.

**Methods:**

Data was used from a two-year longitudinal study. Children wore an accelerometer for up to 8 days 24 h/day across three timepoints. The sample included 2,745 children (51 % girls) between the ages of 7-12-years-old (mean = 9.8 years, SD = 1.1 year) with valid accelerometer data from any timepoint. Reliability estimates were calculated for sleep duration, sleep efficiency, sleep onset, wake time, time in bed, light physical activity, moderate physical activity, moderate-to-vigorous physical activity, vigorous physical activity, and sedentary time.

**Results:**

Intraclass correlations and the Spearman Brown prophecy formula were used to determine the nights and days needed for reliable estimates. We found that between 3 and 5 nights were needed to achieve acceptable reliability (ICC = 0.7) in sleep outcomes, while physical activity and sedentary time outcomes required between 3 and 4 days.

**Conclusions:**

To obtain reliable estimates, researchers should consider these minimum criteria when designing their studies and prepare strategies to ensure sufficient wear time compliance.

**Supplementary Information:**

The online version contains supplementary material available at 10.1186/s12966-021-01143-6.

## Introduction

Accelerometers are valuable devices for measuring free-living movement behaviours, including sleep [1], physical activity, and sedentary time [2]. These devices can provide detailed information about 24-h behaviour across several days, are feasible for large-scale studies, and are less prone to biases and error compared to time-use-diaries which require participant recall [3–6]. Consequently, accelerometers are frequently used in movement behaviour research [7]. While estimates for the number of days to achieve reliable physical activity and sedentary time estimates are generally known [8–13], few studies have examined how many days are needed to reliably estimate habitual sleep using accelerometers [14–16].

Most studies examining how many days are required to estimate habitual physical activity report that 2–7 days are appropriate depending on the activity intensity, type of accelerometer, and position of wear [13]. Some studies also indicate that one or two weekend days are required as well [8–12]. Still, a recent review of accelerometers used in youth physical activity studies showed a wide range of criteria have been applied in this research. The review reported eight different minimum wear day criteria ranging between 1 and 10 days have been used [13]. However, a rough consensus appears to be a 7-day protocol to achieve a 4-day minimum of valid days of physical activity and sedentary time.

The few studies that have reported reliabilities for sleep outcomes in children show that sleep variables may typically need more days than physical activity outcomes to achieve acceptable reliability. Ridgers et al. [15], using the Sensewear armband worn on the upper arm, found the 6 and 7 nights were needed to achieve moderate reliability in sleep duration and time in bed in 8-11-year-old children. Taylor et al. [16], reported 4–7 nights were needed to achieve moderate reliability for sleep duration, sleep efficiency, sleep onset, and wake time for 7-year-old children when employing actigraphy on the hip. Meanwhile, Acebo et al. [14], reported that 3–6 nights are acceptable for 5-year-olds for the same sleep outcomes when measured using actigraphy on the wrist.

Many types of accelerometers currently exist, and researchers need to make informed decisions about which device to use. Among the many considerations is how long participants need to wear the device to get reliable estimates of habitual activity. For example, researchers using accelerometers to determine if children are, on average, meeting 24-h movement guidelines [17] need to know how many days to collect data to make a reliable estimation of typical movement behaviour. In the absence of reliability scores for a specific device, researchers often rely on the procedures of others who have used devices with similar characteristics (i.e., wear location, triaxial, raw acceleration data, etc.) [18]. Ideally, however, reliability scores should be determined for each individual device [4].

Compared to the physical activity and sedentary time research, there is no consensus about the minimum measurement protocol for sleep estimates. In addition, physical activity studies use the number of wear hours as inclusion criteria to determine a valid day. However, none of the sleep studies applied inclusion criteria to their sleep data, instead considering a night valid when there is data recorded [14, 15]. The lack of specific inclusion criteria applied to sleep data may influence how many nights are needed for reliable estimates. Finally, there are no studies that have examined the reliability of sleep estimates using the GENEActiv wrist worn accelerometer which is a relatively new but increasingly popular device amongst movement behaviours researchers [19]. Therefore, given the limited and varied findings for reliabilities of sleep outcomes, the lack of inclusion criteria applied to sleep estimates in previous research, and the unknown reliability of using the GENEActiv accelerometer in children, further examination of the reliability of sleep and physical activity estimates in children is warranted.

The purpose of this study was to investigate the optimal number of nights and valid percentage per night needed to obtain reliable estimates of habitual sleep behaviour (i.e., sleep duration, sleep efficiency, time in bed, sleep onset, and wake time) using accelerometry in children. We also investigated the number of days and hours per day needed to obtain reliable estimates of habitual weekly physical activity and sedentary time.

## Methods

### Participants

Our data comes from the ‘Internet-based Professional Learning to help teachers support Activity in Youth’ (iPLAY) cluster randomized controlled trial [20]. We collected data from primary school children starting in Grade 3 and 4 with follow-up data collection in the following two years (i.e., one-year follow-up and two-year follow-up). For each data collection, the participants wore an accelerometer for eight days. The initial sample included 1,217 children at baseline, 1,027 children at one-year follow-up, and 925 children at two-year follow-up for a total of 3,169 observations or a possible 25,352 monitored days. The Australian Catholic University Research Ethics Committee approved the study (Approval # 2014 185 N) and we obtained written consent from all parents/guardians prior to participation. We collected data between July 2016 and December 2019.

### Accelerometer data

We assessed daily sleep and physical activity using the wrist worn GENEActiv triaxial accelerometer (Activinsights, Cambridge, United Kingdom). We distributed accelerometers to consenting students during their school day and asked teachers to collect the devices immediately after the scheduled monitoring period (i.e., eight days). We asked participants to only remove the accelerometer during contact sports when the device could be a risk of injury, otherwise that the device should be worn on their non-dominant wrist 24 h/day. We set the accelerometers to sample at a frequency of 87.5 Hz and data were stored in 5-second epochs.

We extracted the accelerometer data using the GENEActiv PC Software (ver. 3.3) and processed and analysed the data using the R-package GGIR (ver. 1.10-7) [21] in the R environment (ver. 3.6.1) [22]. GGIR was developed for GENEActiv accelerometers and uses raw acceleration ENMONZ values (i.e., Euclidian norm minus one with negative values set to zero) with validated cut-points to determine intensity of physical activity [23, 24]. We measured the following physical activity variables: sedentary activity (0-56.3 mg), light-intensity physical activity (56.3-191.6 mg), moderate-intensity physical activity (191.6-695.8 mg), vigorous-intensity physical activity (greater than 695.8 mg), and moderate-to-vigorous physical activity (greater than 191.6 mg).

For sleep detection, GGIR identifies periods of sustained inactivity where there is a smaller change in arm angle than a predefined threshold [25]. In this study, we defined the threshold parameters as a change in arm angle of five degrees over a five-minute period. These thresholds have shown good accuracy for sleep detection without the use of an activity diary compared to polysomnography, the gold-standard sleep measure [26]. We measured the following sleep variables: sleep duration, sleep efficiency, time in bed, sleep onset, and wake time.

GGIR also estimates non-wear time for periods of sustained low acceleration. This is determined by the characteristics of 15-min blocks within a 60-min window or by the value range of raw acceleration. That is, blocks are classified as non-wear time when the standard deviation of a window is less than 13 mg or the value range is less than 50 mg for at least two of the three axes of acceleration. GGIR can then impute this missing data based on average ENMONZ values from similar timepoints on other days. GGIR provides two estimates to determine valid wear time: number of valid hours and fraction of the night invalid (%). In this study, we converted the fraction night invalid variable to reflect percentage night valid (e.g., we converted fraction night invalid = 0.25 to 75 % valid). We used valid hours and percentage night valid as criteria for our reliability scores to present ranges of reliability when including 1–24 h/day of valid data for physical activity variables and 50–100 % valid nights for sleep variables. We included all returned accelerometers with extractable data in the analysis.

### Statistical analysis

We conducted all analyses using R (ver. 3.6.3) [22]. To assess reliability, we calculated intraclass correlation coefficients (ICC2) using two-way mixed effects, absolute agreement, single measurement models [27] using the R-package psych [28] for all included variables (see supplementary material for an excerpt of the R code used in the analysis). The ICC is a common method to assess the agreement of measures ranging from 0 to 1.0 where 1.0 indicates perfect reliability or that the variation is all between-subject variation and not within-subjects. ICC values less than 0.5, between 0.5 and 0.75, between 0.75 and 0.9, and greater than 0.9 can be interpreted as poor, moderate, good, and excellent reliability, respectively [29].

We calculated single measurement ICC values, or single day ICC values, for all combinations of inclusion criteria (i.e., valid hours/day for physical activity variables and percentage valid/night for sleep variables). That is, for physical activity, we calculated a single day ICC for each hourly increment starting at a minimum of 1 h to a maximum of 24 h of valid wear time for every two, three, four, five, six, and seven valid days of data. We then randomly sampled days from participants meeting each combination of these criteria. For example, to calculate the single day ICC value for the criteria of 10 h/day of valid wear time, we calculated six ICC values from two randomly sampled days up to seven randomly sampled days (i.e., we included all participants with 10 h/day on at least two days, all participants with 10 h/day on at least three days, etc.) and calculated the average value. In addition, because the days were randomly sampled which resulted in slight variations in ICC values, we repeated the random sampling five times and used the overall average value as the final single day ICC for 10 h/day. This method, which was repeated for each combination of criteria and each physical activity variable, has been used previously for other recent reliability studies [15, 30]. For sleep variables, we used a similar process to calculate ICC values; however, instead of valid wear time hours/day, we used six criteria for percentage night valid from 50, 60, 70, 80, 90, and 100 % from 2 to 7 randomly sampled days of valid data.

We then used the single day ICC values with the Spearman-Brown prophecy formula to determine the number of valid measurement days needed to obtain reliability scores of 0.7, 0.8, and 0.9 [31].

## Results

### Preliminary analyses

**Fig. 1:**
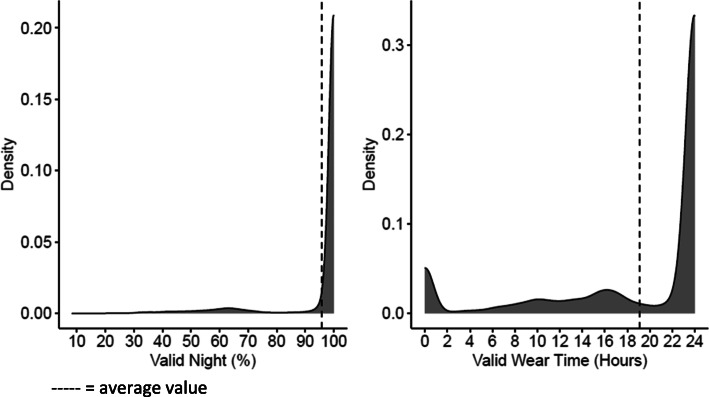
Density plots of the distributions for the sleep criterion percentage night valid (left) and the physical activity criterion for valid wear time (right)

First, we checked the data for calibration errors and device malfunction. We excluded 114 cases (3.9 %) due to accelerometer calibration errors, which indicated that the accelerometer did not record data. These instances occurred primarily at baseline and we removed these accelerometers for follow up data collections. We excluded an additional 13 cases due to extreme outliers (i.e., 3*interquartile range +/- upper/lower quartile) for all outcome variables or for ENMONZ values of acceleration suggesting device error (i.e., malfunctioning). We present the distributions of sleep and physical activity outcomes in supplementary Figure S1. The final sample consisted of 2,745 children (51 % girls) between the ages of 7-12-years-old (mean = 9.8 years, SD = 1.1 year) with at least one day of valid accelerometer data. We then examined the valid wear time and percentage night valid criteria. We show the density plots for each criterion variable in Fig. 1. Our sample showed very good accelerometer wear time compliance. The average wear time/day was 19.1 h (SD = 7.9 h) and the average percentage night valid was 95.8 % (SD = 1.3 %).

### Physical activity and sedentary time outcomes

We present the single day ICC values, number of days needed to achieve 0.7, 0.8, and 0.9 reliability scores for the physical activity and sedentary time outcomes, and the number of participants in our sample that met reliabilities of 0.7 and 0.8 in Table 1. There was little variation in single day ICC values across all valid wear time criteria for each outcome. Single day ICC values only consistently improved with increased wear time for vigorous physical activity and, for the other variables, tended to be the weakest by a small margin between 13 and 16 h of wear time.

**Table 1:** ICC values and number of days needed to achieve 0.7, 0.8, and 0.9 reliability estimates for physical activity and sedentary time outcomes

Minimum Wear Time Criteria (Hours)	Single Day ICC		Number of days to achieve reliabilities of			Number (%) of children meeting reliabilities of	
		95% CI	0.7	0.8	0.9	0.7	0.8
**Light Physical Activity**							
1	0.47	0.45, 0.49	2.6	4.5	10.1	2646 (96.4)	2475 (90.2)
2	0.47	0.45, 0.49	2.6	4.5	10.2	2646 (96.4)	2475 (90.2)
3	0.47	0.45, 0.49	2.6	4.5	10.1	2639 (96.1)	2472 (90.1)
4	0.47	0.45, 0.49	2.6	4.5	10.2	2639 (96.1)	2472 (90.1)
5	0.47	0.45, 0.49	2.7	4.5	10.2	2639 (96.1)	2471 (90.0)
6	0.47	0.45, 0.49	2.6	4.5	10.2	2639 (96.1)	2469 (89.9)
7	0.46	0.44, 0.48	2.7	4.7	10.5	2639 (96.1)	2469 (89.9)
8	0.45	0.43, 0.47	2.8	4.8	10.9	2639 (96.1)	2468 (89.9)
9	0.44	0.42, 0.46	3.0	5.1	11.5	2639 (96.1)	2332 (85.0)
10	0.44	0.42, 0.46	3.0	5.1	11.4	2639 (96.1)	2332 (85.0)
11	0.44	0.42, 0.46	2.9	5.0	11.3	2637 (96.1)	2466 (89.8)
12	0.44	0.43, 0.46	2.9	5.0	11.3	2637 (96.1)	2466 (89.8)
13	0.44	0.42, 0.46	3.0	5.1	11.4	2637 (96.1)	2332 (85.0)
14	0.44	0.42, 0.46	3.0	5.2	11.6	2637 (96.1)	2332 (85.0)
15	0.43	0.41, 0.45	3.1	5.2	11.8	2565 (93.4)	2332 (85.0)
16	0.43	0.41, 0.45	3.1	5.3	11.9	2564 (93.4)	2330 (84.9)
17	0.43	0.41, 0.45	3.0	5.2	11.7	2635 (96.0)	2330 (84.9)
18	0.44	0.42, 0.46	3.0	5.1	11.5	2634 (96.0)	2329 (84.8)
19	0.44	0.42, 0.46	3.0	5.1	11.5	2634 (96.0)	2329 (84.8)
20	0.45	0.43, 0.47	2.9	5.0	11.2	2634 (96.0)	2463 (89.7)
21	0.45	0.43, 0.47	2.8	4.9	11.0	2634 (96.0)	2462 (89.7)
22	0.45	0.43, 0.47	2.8	4.9	10.9	2634 (96.0)	2462 (89.7)
23	0.46	0.43, 0.48	2.8	4.8	10.7	2633 (95.9)	2462 (89.7)
24	0.46	0.44, 0.48	2.8	4.7	10.6	2633 (95.9)	2462 (89.7)
**Moderate Physical Activity**							
1	0.47	0.45, 0.49	2.6	4.5	10.2	2649 (96.5)	2479 (90.3)
2	0.47	0.45, 0.49	2.6	4.5	10.1	2649 (96.5)	2479 (90.3)
3	0.47	0.45, 0.49	2.7	4.5	10.2	2642 (96.2)	2476 (90.2)
4	0.46	0.45, 0.48	2.7	4.6	10.4	2642 (96.2)	2476 (90.2)
5	0.46	0.45, 0.48	2.7	4.6	10.4	2642 (96.2)	2475 (90.2)
6	0.46	0.44, 0.48	2.7	4.7	10.5	2642 (96.2)	2473 (90.1)
7	0.46	0.44, 0.48	2.8	4.7	10.6	2642 (96.2)	2473 (90.1)
8	0.45	0.43, 0.47	2.8	4.9	11.0	2642 (96.2)	2472 (90.1)
9	0.45	0.43, 0.46	2.9	5.0	11.2	2642 (96.2)	2472 (90.1)
10	0.45	0.43, 0.46	2.9	5.0	11.2	2642 (96.2)	2472 (90.1)
11	0.44	0.42, 0.46	3.0	5.1	11.5	2640 (96.2)	2337 (85.1)
12	0.43	0.42, 0.45	3.0	5.2	11.7	2640 (96.2)	2337 (85.1)
13	0.43	0.41, 0.45	3.1	5.3	11.9	2567 (93.5)	2337 (85.1)
14	0.43	0.41, 0.45	3.1	5.3	11.9	2567 (93.5)	2337 (85.1)
15	0.43	0.41, 0.45	3.1	5.3	12.0	2567 (93.5)	2337 (85.1)
16	0.43	0.41, 0.45	3.1	5.3	11.8	2566 (93.5)	2335 (85.1)
17	0.43	0.41, 0.45	3.0	5.2	11.7	2638 (96.1)	2335 (85.1)
18	0.44	0.42, 0.46	3.0	5.1	11.5	2637 (96.1)	2334 (85.0)
19	0.44	0.42, 0.46	2.9	5.0	11.4	2637 (96.1)	2467 (89.9)
20	0.44	0.42, 0.46	3.0	5.1	11.5	2637 (96.1)	2334 (85.0)
21	0.44	0.42, 0.46	2.9	5.0	11.3	2637 (96.1)	2466 (89.8)
22	0.44	0.42, 0.47	2.9	5.0	11.3	2637 (96.1)	2466 (89.8)
23	0.44	0.42, 0.46	3.0	5.1	11.4	2636 (96.0)	2332 (85.0)
24	0.44	0.42, 0.46	2.9	5.0	11.4	2636 (96.0)	2466 (89.8)
**Moderate to Vigorous Physical Activity**							
1	0.46	0.45, 0.48	2.7	4.6	10.4	2649 (96.5)	2479 (90.3)
2	0.46	0.44, 0.48	2.7	4.7	10.5	2649 (96.5)	2479 (90.3)
3	0.46	0.44, 0.48	2.7	4.7	10.5	2642 (96.2)	2476 (90.2)
4	0.46	0.45, 0.48	2.7	4.6	10.4	2642 (96.2)	2476 (90.2)
5	0.46	0.44, 0.48	2.7	4.7	10.6	2642 (96.2)	2475 (90.2)
6	0.46	0.44, 0.48	2.8	4.7	10.6	2642 (96.2)	2473 (90.1)
7	0.45	0.44, 0.47	2.8	4.8	10.8	2642 (96.2)	2473 (90.1)
8	0.45	0.43, 0.47	2.8	4.9	10.9	2642 (96.2)	2472 (90.1)
9	0.45	0.43, 0.47	2.9	5.0	11.1	2642 (96.2)	2472 (90.1)
10	0.44	0.42, 0.46	3.0	5.1	11.4	2642 (96.2)	2337 (85.1)
11	0.44	0.42, 0.46	3.0	5.1	11.6	2640 (96.2)	2337 (85.1)
12	0.43	0.41, 0.45	3.1	5.3	11.8	2568 (93.6)	2337 (85.1)
13	0.43	0.41, 0.45	3.1	5.3	11.9	2567 (93.5)	2337 (85.1)
14	0.43	0.41, 0.45	3.1	5.3	12.0	2567 (93.5)	2337 (85.1)
15	0.43	0.41, 0.45	3.1	5.2	11.8	2567 (93.5)	2337 (85.1)
16	0.43	0.41, 0.45	3.1	5.3	11.9	2566 (93.5)	2335 (85.1)
17	0.44	0.42, 0.46	3.0	5.1	11.6	2638 (96.1)	2335 (85.1)
18	0.44	0.42, 0.46	3.0	5.1	11.4	2637 (96.1)	2334 (85.0)
19	0.44	0.42, 0.46	2.9	5.0	11.3	2637 (96.1)	2467 (89.9)
20	0.44	0.42, 0.46	3.0	5.1	11.4	2637 (96.1)	2334 (85.0)
21	0.44	0.42, 0.46	2.9	5.0	11.3	2637 (96.1)	2466 (89.8)
22	0.45	0.43, 0.47	2.9	5.0	11.2	2637 (96.1)	2466 (89.8)
23	0.44	0.42, 0.46	3.0	5.1	11.4	2636 (96.0)	2332 (85.0)
24	0.44	0.42, 0.47	2.9	5.0	11.3	2636 (96.0)	2466 (89.8)
**Vigorous Physical Activity**							
1	0.39	0.37, 0.41	3.6	6.2	14.0	2570 (93.6)	2144 (78.1)
2	0.39	0.37, 0.41	3.6	6.2	14.0	2570 (93.6)	2144 (78.1)
3	0.39	0.37, 0.41	3.6	6.2	13.9	2569 (93.6)	2142 (78.0)
4	0.40	0.38, 0.41	3.6	6.1	13.7	2569 (93.6)	2142 (78.0)
5	0.39	0.37, 0.41	3.6	6.2	14.0	2569 (93.6)	2142 (78.0)
6	0.39	0.37, 0.41	3.6	6.2	13.9	2569 (93.6)	2139 (77.9)
7	0.39	0.38, 0.41	3.6	6.1	13.8	2569 (93.6)	2139 (77.9)
8	0.39	0.37, 0.41	3.6	6.2	13.9	2569 (93.6)	2129 (77.6)
9	0.39	0.37, 0.41	3.6	6.2	14.0	2569 (93.6)	2129 (77.6)
10	0.40	0.38, 0.42	3.5	6.1	13.7	2569 (93.6)	2129 (77.6)
11	0.40	0.39, 0.42	3.4	5.9	13.3	2568 (93.6)	2328 (84.8)
12	0.41	0.39, 0.43	3.4	5.9	13.2	2568 (93.6)	2328 (84.8)
13	0.41	0.39, 0.43	3.4	5.8	13.0	2567 (93.5)	2328 (84.8)
14	0.42	0.40, 0.44	3.3	5.6	12.6	2567 (93.5)	2328 (84.8)
15	0.42	0.40, 0.44	3.3	5.6	12.6	2567 (93.5)	2328 (84.8)
16	0.42	0.40, 0.44	3.2	5.4	12.2	2566 (93.5)	2326 (84.7)
17	0.43	0.41, 0.45	3.0	5.2	11.7	2637 (96.1)	2326 (84.7)
18	0.44	0.42, 0.46	3.0	5.1	11.4	2636 (96.0)	2325 (84.7)
19	0.44	0.42, 0.46	3.0	5.1	11.6	2636 (96.0)	2325 (84.7)
20	0.44	0.42, 0.46	2.9	5.0	11.3	2636 (96.0)	2462 (89.7)
21	0.44	0.42, 0.46	2.9	5.0	11.3	2636 (96.0)	2461 (89.7)
22	0.45	0.42, 0.47	2.9	5.0	11.2	2636 (96.0)	2461 (89.7)
23	0.44	0.42, 0.46	3.0	5.1	11.4	2635 (96.0)	2323 (84.6)
24	0.44	0.42, 0.46	3.0	5.1	11.4	2635 (96.0)	2323 (84.6)
**Sedentary Time**							
1	0.42	0.40, 0.44	3.2	5.4	12.3	2318 (84.4)	2045 (74.5)
2	0.43	0.41, 0.45	3.1	5.4	12.1	2318 (84.4)	2045 (74.5)
3	0.42	0.40, 0.44	3.2	5.4	12.2	2317 (84.4)	2045 (74.5)
4	0.42	0.41, 0.44	3.2	5.4	12.2	2317 (84.4)	2045 (74.5)
5	0.43	0.41, 0.45	3.1	5.3	12.0	2317 (84.4)	2045 (74.5)
6	0.43	0.41, 0.45	3.1	5.3	12.0	2317 (84.4)	2045 (74.5)
7	0.43	0.41, 0.44	3.2	5.4	12.2	2317 (84.4)	2045 (74.5)
8	0.43	0.41, 0.45	3.1	5.4	12.0	2317 (84.4)	2043 (74.4)
9	0.42	0.40, 0.44	3.2	5.5	12.3	2317 (84.4)	2043 (74.4)
10	0.42	0.40, 0.44	3.2	5.6	12.5	2317 (84.4)	2043 (74.4)
11	0.41	0.39, 0.43	3.4	5.8	13.0	2317 (84.4)	2043 (74.4)
12	0.40	0.38, 0.42	3.4	5.9	13.3	2317 (84.4)	2043 (74.4)
13	0.40	0.38, 0.42	3.5	6.0	13.6	2316 (84.4)	2042 (74.4)
14	0.39	0.37, 0.41	3.6	6.3	14.1	2316 (84.4)	1805 (65.8)
15	0.37	0.35, 0.39	4.0	6.8	15.3	2316 (84.4)	1805 (65.8)
16	0.35	0.33, 0.37	4.3	7.4	16.6	2191 (79.8)	1114 (40.6)
17	0.35	0.33, 0.37	4.4	7.5	16.8	2191 (79.8)	1114 (40.6)
18	0.36	0.34, 0.38	4.1	7.0	15.8	2191 (79.8)	1805 (65.8)
19	0.37	0.35, 0.40	3.9	6.7	15.1	2316 (84.4)	1805 (65.8)
20	0.38	0.36, 0.40	3.8	6.5	14.6	2316 (84.4)	1805 (65.8)
21	0.39	0.36, 0.41	3.7	6.4	14.3	2316 (84.4)	1805 (65.8)
22	0.39	0.37, 0.41	3.6	6.2	14.0	2316 (84.4)	1805 (65.8)
23	0.40	0.37, 0.42	3.6	6.1	13.7	2316 (84.4)	1805 (65.8)
24	0.41	0.38, 0.43	3.4	5.8	13.1	2316 (84.4)	2039 (74.3)

To achieve acceptable reliabilities of 0.7 and 0.8 for light, moderate, and moderate-to-vigorous physical activity, 3–4 and 5–6 days were needed, respectively. Similarly, vigorous physical activity required 3–4 days to achieve a reliability of 0.7 but needed 5–7 days to achieve 0.8. Sedentary time required the most days, needing 4–5 days and 6–8 days for reliabilities of 0.7 and 0.8, respectively.

Nearly all of the sample met criteria for moderate reliability of 0.7 across all minimum wear time hours (i.e., light physical activity 93.4–96.4 %, moderate physical activity 93.5–96.5 %, moderate-to-vigorous physical activity 93.5–96.5 %, vigorous physical activity 93.5–96.0 %, and sedentary time 79.8–84.4 %), indicating high wear time compliance within the sample.

### Sleep outcomes

We present the single day ICC values, number of days needed to achieve 0.7, 0.8, and 0.9 reliability scores for sleep outcomes, and the number of participants in our sample that met reliabilities of 0.7 and 0.8 in Table 2. The was little variation between the lowest percentage night valid and the highest; however, the single day ICC values tended to increase slightly as the criteria increased for all sleep outcomes.

**Table 2:** ICC values and number of days needed to achieve 0.7, 0.8, and 0.9 reliability estimates for sleep outcomes

Night Valid Criteria (%)	Single Day ICC		No. of days to achieve reliabilities of			No. (%) of children meeting reliabilities of	
		95% CI	0.7	0.8	0.9	0.7	0.8
**Sleep Duration**							
50	0.33	0.31, 0.35	4.7	8.0	18.0	2094 (76.3)	1022 (37.2)
60	0.34	0.32, 0.36	4.5	7.6	17.2	2063 (75.2)	1002 (36.5)
70	0.34	0.32, 0.36	4.5	7.7	17.4	2027 (73.8)	970 (35.3)
80	0.34	0.32, 0.36	4.5	7.7	17.4	2013 (73.3)	953 (34.7)
90	0.35	0.33, 0.37	4.4	7.5	16.8	1995 (72.7)	922 (33.6)
100	0.36	0.34, 0.38	4.1	7.1	16.0	1849 (67.4)	515 (18.8)
**Sleep Efficiency**							
50	0.41	0.39, 0.43	3.4	5.8	13.0	2152 (78.4)	1891 (68.9)
60	0.41	0.39, 0.43	3.3	5.7	12.8	2140 (78.0)	1881 (68.5)
70	0.42	0.40, 0.44	3.2	5.4	12.2	2126 (77.4)	1870 (68.1)
80	0.43	0.41, 0.45	3.1	5.3	12.0	2120 (77.2)	1863 (67.9)
90	0.44	0.42, 0.46	3.0	5.1	11.5	2214 (80.7)	1849 (67.4)
100	0.46	0.44, 0.48	2.7	4.6	10.4	2153 (78.4)	1848 (67.3)
**Time in Bed**							
50	0.33	0.31, 0.35	4.7	8.0	18.1	2097 (76.4)	1082 (39.4)
60	0.34	0.32, 0.36	4.5	7.6	17.2	2080 (75.8)	1072 (39.1)
70	0.35	0.33, 0.37	4.3	7.4	16.6	2045 (74.5)	1044 (38.0)
80	0.36	0.34, 0.38	4.2	7.2	16.1	2031 (74.0)	1031 (37.6)
90	0.36	0.34, 0.38	4.1	7.0	15.8	2012 (73.3)	1643 (59.9)
100	0.37	0.35, 0.40	3.9	6.7	15.1	2025 (73.8)	1219 (44.4)
**Sleep Onset**							
50	0.43	0.41, 0.45	3.1	5.4	12.1	2034 (74.1)	1792 (65.3)
60	0.42	0.40, 0.44	3.3	5.6	12.6	2011 (73.3)	1775 (64.7)
70	0.42	0.40, 0.44	3.3	5.6	12.6	1952 (71.1)	1731 (63.1)
80	0.42	0.40, 0.44	3.2	5.5	12.3	1941 (70.7)	1714 (62.4)
90	0.42	0.40, 0.45	3.2	5.4	12.2	1931 (70.3)	1698 (61.9)
100	0.44	0.42, 0.46	3.0	5.1	11.5	1964 (71.5)	1478 (53.8)
**Wake Time**							
50	0.33	0.31, 0.35	4.7	8.1	18.1	1928 (70.2)	-
60	0.33	0.31, 0.36	4.6	8.0	17.9	1894 (69.0)	951 (34.6)
70	0.33	0.31, 0.36	4.7	8.0	18.0	1839 (67.0)	928 (33.8)
80	0.34	0.32, 0.36	4.5	7.7	17.3	1826 (66.5)	915 (33.3)
90	0.34	0.32, 0.37	4.5	7.6	17.2	1812 (66.0)	887 (32.3)
100	0.35	0.32, 0.37	4.4	7.6	17.0	1672 (60.9)	500 (18.2)

Sleep duration and wake time for all percent night valid criteria required 5 and 8 nights to achieve reliabilities of 0.7 and 0.8, respectively. Time in bed with 100 % night valid needed 4 and 7 nights to achieve acceptable reliabilities. For sleep onset to achieve acceptable reliabilities of 0.7 and 0.8, 4 and 6 nights were needed but at 100 % night valid 3 days were enough at the 0.7 level. We found sleep efficiency needed the least nights of all the sleep outcomes, requiring 3 nights with 90 % or more night valid and 5 nights with 100 % valid data to achieve reliabilities of 0.7 and 0.8, respectively. Across all sleep outcomes, the range of participants meeting criteria for a reliability of 0.7 was 60.9–80.7 %.

## Discussion

The purpose of this study was to investigate the numbers nights needed to achieve reliable estimates sleep duration, sleep efficiency, time in bed, sleep onset, and wake time in children using the GENEActiv accelerometer. We also investigated the number of days that needed to reliably estimate habitual light physical activity, moderate physical activity, moderate-to-vigorous physical activity, vigorous physical activity, and sedentary time. We found that the numbers of days needed to obtain reliable estimates varied by outcome variable and by inclusion criteria. Broadly, we found that 4 days, for almost all valid hour criteria, would be enough to achieve moderate reliability (i.e., 0.7) for all physical activity and sedentary time outcomes. For moderately reliable estimates of habitual sleep behaviour, we found that 5 nights are needed.

There was little variation in single day ICC values across minimum valid hour criteria in physical activity variables. Other studies have shown a pattern whereby increased wear time criteria resulted in larger ICC values [15, 32]. Meaning that increased valid hours required less days to achieve acceptable reliability. In our study, only vigorous physical activity showed this pattern. Still, our ICC values tended to be similar in size to previous studies [15, 32] and overall our findings for physical activity fit the general consensus that 4 days of valid data are needed for reliable estimates [13]. Sedentary time tended to have lower ICC values compared to the physical activity outcomes, indicating that there is more variability in sedentary time across days which resulted in 4–5 days being required. This is more days than reported by Dillon et al. [11] but similar to other studies [9, 33].

Single day ICC values for sleep outcomes increased in size as percent night valid criteria became more stringent; however, sleep ICC values tended to be smaller than those for physical activity. Consequently, more nights are needed for reliable estimates of habitual sleep behaviour than for habitual physical activity. Sleep efficiency and sleep onset showed stronger ICC values (range = 0.41–0.46) compared to sleep duration, time in bed, and wake time (range = 0.33–0.37). Our findings for sleep duration and time in bed required less days to achieve acceptable reliability (i.e., 4–5 nights) compared to Ridgers et al. [15] and Acebo et al. [14], who reported 6–7 nights are needed for sleep duration and time in bed. Sleep efficiency and sleep onset also needed fewer nights (i.e., 3–4 nights) than has been stated by Taylor et al. [16], who reported 4–7 nights are needed. Wake time in our study, however, required more nights (i.e., 5 nights compared to 2–4 nights). For all sleep outcomes, the reliability was best when using the valid night criteria of 100 %.

Children in our sample were less likely to have valid sleep data and meet criteria for reliable data compared to the physical activity outcomes. For example, for reliabilities of 0.7, most of the sample met even the most stringent wear time criterion (i.e., 24 h/day wear time) with between 84.4 and 96 % of children included. Sleep, on the other hand, under the same most stringent criteria (i.e., 100 % valid night data) included 60.9–78.4 % of the sample. Furthermore, even at a reliability of 0.8 most children with 24 h/day of wear time were still included (i.e., 74.3 % for sedentary time to 89.8 % for moderate to vigorous physical activity). However, sample sizes for some sleep outcomes drop considerably when 100 % valid night criteria and reliabilities of 0.8 were considered (i.e., 18.8 % for sleep duration and 18.1 % for wake time). Researchers need to consider the effect that level of reliability and movement behaviour inclusion criteria have on sample size when considering their measurement protocols. The reliability criterion of 0.7 is widely used and considered acceptable for this research to both reduce participant burden and maximise participant retention [34].

There was very little difference in physical activity ICC values from 1 to 24 h (e.g., ICC for at least 1 h/day light physical activity was 0.47 while 24 h/day was 0.46). This may be due to the high wear time compliance in our sample which resulted in little variation at lower hours of wear time. Regardless, we do not recommend that one hour of wear time is sufficient to estimate habitual physical activity. Rather, we have presented all available data so that researchers can make informed decisions for their study protocols that are founded on evidence and theory. Previous research has commonly used a minimum of 8–10 h to define a valid day [13].

A key strength of our study is the use of sleep inclusion criteria. Other studies have not used an inclusion criterion for their sleep estimates other than “has data”. This is important because it provides an indication of the quality of the sleep estimates being used in the analyses. We have also examined a variety of daily movement behaviours and several dimensions of sleep. Another strength of our study is the large sample size with high accelerometer wear time compliance which potentially provided more generalisable and precise results than smaller studies with poorer wear time. Considering that for children who had 24 h of valid wear time and 100 % valid sleep still required four days and five nights, respectively, indicates that there is considerable variation in children’s day-to-day movement patterns not explained by wear time or accelerometer performance. While we only investigated reliability of one device, we believe these estimated wear times would also hold for other devices (i.e., Actigraph GT3X, Axivity AX3, etc.), given that the devices are wrist-worn, triaxial, and provide raw acceleration data.

Notwithstanding these strengths, one limitation of our study is that we did not specifically examine the inclusion of weekend days. Some studies have reported that weekend days are required for reliable estimates [13], while others have stated that the inclusion of weekend data is not necessary [35]. Our analyses, however, randomly sampled valid days and nights which included weekends. Therefore, our recommendation that, for example, moderate physical activity needs four days of valid data could be any combination of weekday and weekend days. This approach provides a less stringent inclusion criterion by simply requiring a certain number of days regardless of which days. A recent study found no difference between week and weekend physical activity in children [36], which supports using this method. However, the study found differences in adults meaning that, while our findings may be generalisable to other children, they may not be suitable for studies among adults. Another limitation is that the window of time that these estimates reliably predict is unknown. That is, five days of sleep behaviour data provides a reliable estimate of habitual sleep for a given week, but we do not know if it is reliable for a month or longer. More research is needed to determine the measurement protocol needed to estimate longer periods of habitual activity.

## Conclusions

Our study examined the number of nights and days needed to reliably estimate sleep, physical activity, and sedentary time using the GENEActiv accelerometer. The findings from our study suggest that 5 nights of valid sleep data would provide acceptable reliability for habitual sleep behaviour. We also found that at least 4 days of valid data would provide acceptable reliability for habitual physical activity and sedentary time, across all minimum daily wear time criteria. Researchers should account for the effect that various inclusion criteria may have on study sample size and consider adjustments to their study designs or strategies for ensuring sufficient wear time compliance to achieve an acceptable level of reliability.

## Supplementary Information

**Additional file 1: Supplementary Figure S1.** Distributions of sleep and physical activity variables.

**Additional file 2.**

## Data Availability

The datasets used and/or analysed during the current study are available from the corresponding author on reasonable request.
