# Supplementary figures and images for: Reliability of GENEActiv accelerometers to estimate sleep, physical activity, and sedentary time in children
Source: Int J Behav Nutr Phys Act. 2021 Jun 6;18:73. doi: 10.1186/s12966-021-01143-6 (PMC8180134; doi:10.1186/s12966-021-01143-6)

**Supplementary Figure S1** Distributions of sleep and physical activity variables.


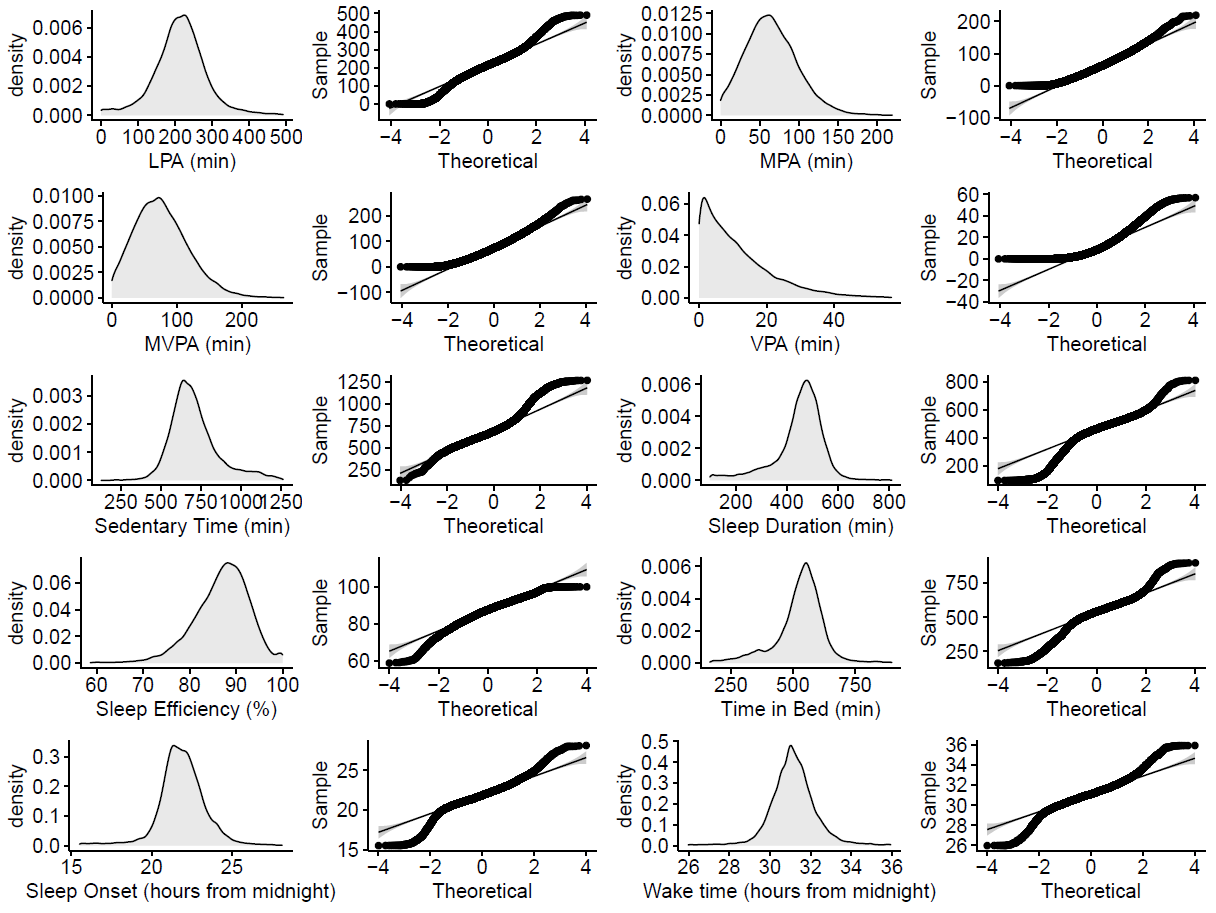

Supplement: Supplementary file 1 — Additional file 1: Supplementary Figure S1. Distributions of sleep and physical activity variables. [file 12966_2021_1143_MOESM1_ESM.docx]
